# Prognostic Value of Plasma Catestatin Concentration in Patients with Heart Failure with Reduced Ejection Fraction in Two-Year Follow-Up

**DOI:** 10.3390/jcm12134208

**Published:** 2023-06-22

**Authors:** Łukasz Wołowiec, Joanna Banach, Jacek Budzyński, Anna Wołowiec, Mariusz Kozakiewicz, Maciej Bieliński, Albert Jaśniak, Agata Olejarczyk, Grzegorz Grześk

**Affiliations:** 1 Department of Cardiology and Clinical Pharmacology, Faculty of Health Sciences, Collegium Medicum in Bydgoszcz, Nicolaus Copernicus University, 87-100 Toruń, Polandalbertjasniak@gmail.com (A.J.); ggrzesk@cm.umk.pl (G.G.); 2 Department of Vascular and Internal Diseases, Faculty of Health Sciences, Collegium Medicum in Bydgoszcz, Nicolaus Copernicus University, 87-100 Toruń, Poland; jb112233@cm.umk.pl (J.B.); agatuh@gmail.com (A.O.); 3 Department of Geriatrics, Division of Biochemistry and Biogerontology, Faculty of Health Sciences, Collegium Medicum in Bydgoszcz, Nicolaus Copernicus University, 87-100 Toruń, Poland; anna.wolowiec@cm.umk.pl (A.W.); markoz@cm.umk.pl (M.K.); 4 Department of Clinical Neuropsychology, Faculty of Health Sciences, Collegium Medicum in Bydgoszcz, Nicolaus Copernicus University, 85-094 Bydgoszcz, Poland; maciejb@cm.umk.pl

**Keywords:** catestatin, heart failure, HFrEF, biomarker, biomarkers for heart failure prognosis

## Abstract

The primary objective of the study was to evaluate the prognostic value of measuring plasma catestatin (CST) concentration in patients with heart failure with reduced ejection fraction (HFrEF) as a predictor of unplanned hospitalization and all-cause death independently and as a composite endpoint at 2-year follow-up. The study group includes 122 hospitalized Caucasian patients in NYHA classes II to IV. Patients who died during the 24-month follow-up period (*n* = 44; 36%) were significantly older on the day of enrollment, were more likely to be in a higher NYHA class, had lower TAPSE, hemoglobin concentration, hematocrit, and platelet count, higher concentrations of CST, NT-proBNP, troponin T, creatinine, and glucose, and higher red cell distribution width value and leukocyte and neutrocyte count than patients who survived the follow-up period. Plasma catestatin concentration increased with NYHA class (R = 0.58; *p* <0.001) and correlated significantly with blood NT-proBNP concentration (R = 0.44; *p* <0.001). We showed that higher plasma catestatin concentration increased the risk of all-cause death by more than five times. Plasma CST concentration is a valuable prognostic parameter in predicting death from all causes and unplanned hospitalization in patients with HFrEF.

## 1. Introduction

The prevalence of heart failure (HF) in the general adult population is 1–3% [1] and increases rapidly after the age of 75, reaching 20% in people aged 70–80 [1]. These statistics seem to be constantly increasing, which can be associated with the phenomenon of aging populations and improved survival after acute myocardial infarction. HF is the most common cause of hospitalization in patients >65 years of age [2]. The high rate of expensive readmissions is one of the main causes of the economic burden of HF, which is a major challenge for health care systems worldwide.

The annual health care cost per HF patient in Germany in 2011 was EUR 3150 per year and increases with the severity of the disease according to the New York Heart Association (NYHA) classification of heart failure. Compared to the EUR 2474 spent per patient in NYHA class I, the cost in NYHA class II increases by 14%, in NYHA class III by 48%, and in NYHA class IV by 71% [3]. The cost of hospitalization ranges from 74 to 90% of the total direct and indirect expenditure related to HF [1,3]. In 2012, the American Heart Association estimated a 2.5-fold increase in the medical costs of HF in the United States, from USD 20.9 billion in 2012 to USD 53.1 billion in 2030, and an approximately 2-fold increase in HF cases in men [4]. These statistics show the great importance of precise risk stratification enabling early identification of subgroups of patients with the worst prognosis in order to quickly implement optimal treatment methods. Due to the important role of markers in HF [5,6,7,8,9,10,11] and the need to discover new ones, we decided to evaluate the clinical usefulness of measuring plasma catestatin (CST) in patients with HFrEF as a predictor of unplanned hospitalization and all-cause death independently and as a composite endpoint at a two-year follow-up.

CST is a bioactive peptide composed of 21 amino acids that is formed as a result of the cleavage of the prohormone chromogranin A (CgA). CgA occurs in the chromaffin cells of the adrenal medulla and in other cells of the neuroendocrine and nervous systems, from where it is secreted from secretory granules by autocrine signaling, or together with catecholamines (CA) by exocytosis. CgA has also been detected in endomyocardial biopsy material in people with heart disease [12], as well as in normal heart tissues where a decrease in CST concentration was observed with age [13]. Unlike CA, which is rapidly degraded in plasma, CST is highly stable and is considered a useful marker of sympathetic activation. Previous clinical studies show that the level of CST can remain elevated for some time despite treatment and alleviation of HF symptoms [14]. 

In 1997, Mahata et al. synthesized 15 peptides that make up CgA and showed that only one of them inhibited nicotine-induced CA secretion [15]. They named the discovered peptide CST because of its high ability to inhibit CA release [16] by binding to nicotinic acetylcholine receptors that block Na+ uptake [15]. A direct antagonistic effect of CST on β-adrenergic receptors with a hypotensive effect has also been described [17]. In humans, plasma CST concentrations have been shown to increase in situations of marked sympathetic stimulation, such as in critically ill patients [18]. High plasma CST concentrations have been found in people with HF [5] due to coronary artery disease [19], hypertension [20], dilated cardiomyopathy [12], hypertrophic cardiomyopathy [12], and valvular disease [12]. Plasma CgA concentrations have been shown to increase with the advancement of various human heart diseases [21].

The main cardioprotective properties of CST can be derived from the in vivo vasodilation observed. The beneficial afterload reduction resulting from vasodilatation appears to be a multifactorial mechanism mediated by histamine, along with a reduction in reactive oxygen species availability and stimulation of nitric oxide production [22]. CST activates the β2-ARs-PI3K-eNOs-NO (β2-adrenergic receptors-phosphoinositide 3-kinases-endothelial nitric oxide synthase-nitric oxide) signaling pathway in endocardial endothelial cells, which play a role in myocardial remodeling. In connection with the above, it can be assumed that the inhibition of the fibrosis process by CST occurs through increased production of NO [23,24]. Another anti-hypertrophic mechanism for modulating coronary vascular tone is blocked by the CST receptor for endothelin-1 [25,26]. Additional mechanisms may include pleiotropism-limiting metabolic syndromes, including research-proven anti-inflammatory, anti-atherosclerotic, anti-diabetic and anti-apoptotic effects. CST suppresses TNF-α-elicited expression of inflammatory cytokines and adhesion molecules by activating angiotensin-converting enzyme 2 (ACE2), [27], induces glucose uptake and Glut4 (Glucose transporter type 4) translocation in cardiomyocytes [28], upregulates insulin-induced Akt phosphorylation, reduces endoplasmic reticulum stress (ERS) and increases insulin sensitivity [29]. CST inhibits ERS-induced cardiomyocyte apoptosis by simultaneously activating the β2-adrenergic receptor and the PKB/Akt (phosphatidylinositol 3-kinase/protein kinase B) pathway [30]. CST supports the immunosuppression of macrophages and differentiates their phenotypes towards anti-inflammatory behavior [31], promotes the oxidation of fatty acids and their flow from adipose tissue to the liver, and lowers plasma leptin levels by resensitizing receptors to leptin [32].

The main aim of our study was to assess the usefulness of determining plasma CST concentration, collected within 24 h of admission to the hospital or first contact with a health professional at a clinic, as a predictor of unplanned hospitalization and all-cause mortality independently and as a CE in the group of patients with HFrEF at a two-year follow-up.

## 2. Materials and Methods

### 2.1. Study Population

The study group consisted of 122 Caucasian patients with HFrEF in NYHA classes II-IV, who were either treated on an outpatient basis or hospitalized in the Department of Cardiology and Clinical Pharmacology or the Department of Vascular and Internal Diseases at the Nicolaus Copernicus University Collegium Medicum University Hospital No. 2 in Bydgoszcz, Poland, for planned medical procedures, some of whom were on the elective list of patients waiting for heart transplantation. Outpatient patients were under the care of the Cardiology Clinic and Heart Failure Clinic operating in the department. The diagnosis of HFrEF was based on the criteria of the European Society of Cardiology (ESC). Patients received optimal pharmacological treatment for each patient in accordance with the ESC guidelines [33].

The study protocol was approved by the Bioethical Committee of the Nicolaus Copernicus University in Toruń at the Collegium Medicum in Bydgoszcz. Each patient signed an informed consent form after obtaining detailed information about the purpose and scope of the study. The criteria for inclusion in the study were age over 18, heart failure diagnosed according to the criteria included in the guidelines of the European Society of Cardiology, heart failure class II-IV according to NYHA, left ventricular ejection fraction (LVEF) ≤ 40% assessed during the current hospitalization or up to 6 months earlier. The exclusion criteria were sepsis or shock from any cause on admission to hospital, acute coronary syndrome, recent (<3 months) myocardial infarction or stroke, active neoplasm, autoimmune diseases, impaired liver function (INR without oral anticoagulation > 1.5, bilirubin total > 1.5 mg%, or 3 times the upper limit of normal for ALT), corticosteroid therapy, decompensated diabetes mellitus requiring treatment with intravenous insulin infusion, chronic inflammatory bowel diseases, or recent (<3 months) surgery.

### 2.2. Catestatin Determination

All biochemical analytes were routinely collected upon admission to the Department of Cardiology and Clinical Pharmacology and Clinical Pharmacology or the Department of Vascular and Internal Diseases. Blood specimens were collected by venipuncture into 5 mL tubes containing tripotassium EDTA. Plasma blood was centrifuged (3000× *g* for 15 min) and aliquoted into Eppendorf tubes. Samples were stored at a temperature of −80 °C until biochemical analysis was performed. The plasma catestatin concentration was measured by an enzyme immunoassay (ELISA) commercial kit, Human Catestatin EIA, from RayBiotech, (Norcross, GA, USA), catalog number EIA-CAT-1, dilution factor 12 × for human, reproducibility intra-assay: CV and < 10%, and inter-assay: CV and < 15%. According to the manufacturer, the reactivity with human CST is 100%. The analytical sensitivity of the method (lower detection limit for the test) is 0.5 ng/mL. The results were obtained with a SPECTROstar Nano (BMG LABTECH) spectrophotometric reader using MARS data analysis software version 2.41. The marker was evaluated at the 450 nm wavelength. The results were read from the calibration curve prepared for the analyzer used in the study. All analyses were performed in accordance with the manufacturer’s instructions.

### 2.3. Statistical Analysis

Statistical analysis was conducted using the licensed version of the statistical analysis software STATISTICA version 13.1 (TIBCO Software, Inc., Palo Alto, CA, USA, 2017). The statistical significance level was set at a *p*-value of < 0.05. The normal distribution of the study variables was analyzed using the Kolmogorov–Smirnov test. The results were presented as the mean ± standard deviation; median, interquartile range (IQR); or as a frequency (*n*, %) of the categorical variables. The statistical significance of differences between groups was verified using the Student’s *t*-test, the Mann–Whitney U-test, and one-factorial ANOVA with Bonferroni post hoc test for quantitative variables (when more than one comparison was necessary) and the Chi-square test for qualitative variables. We also used a ROC curve with the lowest Youden index and the AUC to determine the cut-off values of the parameters measured. Kaplan–Meir analysis and Cox hazards regression model were performed to determine the factors affecting the risk of all-cause mortality and all-cause readmission. Spearman’s correlation was also used. The logistic regression method was applied to determine the risk of measured outcome occurrence associated with respective cut-offs of the parameters studied.

## 3. Results

### 3.1. Clinical Characteriscits

Of 122 consecutive HFrEF patients, 78 were admitted to the hospital with exacerbation (including de novo heart failure) and 44 were in stable condition. Although there is no precise definition of heart failure exacerbation, we decided to include only patients who demonstrated certain dynamics of increasing hypervolaemia, and ultimately urged these patients to seek medical care. Low-output states were excluded from the study, as in our opinion in initial stages it is difficult to make a clear distinction between plain HF exacerbation with hypotension and developing cardiogenic shock, a classic form of acute heart failure. Two patients were not included in the final analysis due to lack of survival data after 12 months of follow-up. Patients who died during the follow-up period (*n* = 44, 36%) were significantly older at the day of enrollment, were more likely to be in a higher NYHA class, and had lower TAPSE, hemoglobin concentration, hematocrit, and platelets count, higher concentrations of catestatin, NT-proBNP, troponin T, creatinine, and glucose, and higher RDW values and leukocyte and neutrocyte counts than patients who survived the follow-up period (Table 1). Pharmacotherapy differed between groups, mainly in relation to ASA (acetylsalicylic acid), NOACs (non-vitamin K oral anticoagulants), and diuretic use (Table 2). 

Plasma catestatin concentration increased with NYHA class (R = 0.58; *p* < 0.001); however, those relationships were weaker than those obtained for NT-proBNP (R = 0.66; *p* < 0.001). Plasma catestatin concentration correlated significantly with blood NT-proBNP concentration (R = 0.44; *p* < 0.001) and did not reach significance for BMI.

### 3.2. Plasma Catestatin Concentration as a Factor for Discriminating among HFrEF Patients

In the next step of analysis, using ROC curve analysis, we determined how catestatin cut-off predicted risk of all-cause mortality during long-term follow up. Compared to patients with lower plasma catestatin concentrations, those with plasma catestatin concentrations higher than or equal to the established cut-off (27.94 pmol/L; AUC, 95% CI: 0.706, 0.609–0.803, *p* < 0.001) were significantly older and had higher prevalence of advanced NYHA class, all-cause readmission, mortality, and composite endpoints, and shorter survival time (Table 2). Statistically significant differences between groups mentioned also included: prevalence of hypertension, history of CIED implantation, TAPSE, NT-proBNP, troponin T, creatinine, glucose, neutrocyte percentage, hemoglobin, and hematocrit (Table 3). Correlations between catestatin and selected parameters are presented in Table 4.

### 3.3. Survival Analysis

**Figure 1:**
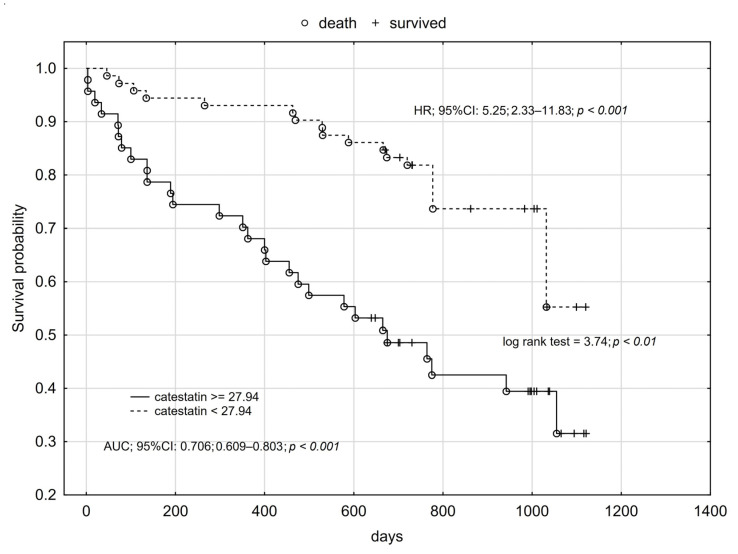
Kaplan–Meier analysis for all-cause mortality of HFrEF patients in relation to plasma catestatin concentration.

**Figure 2:**
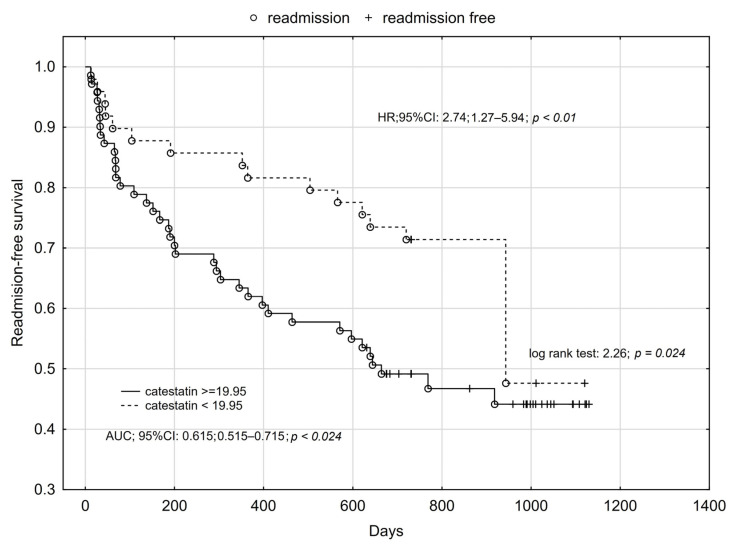
Kaplan–Meier analysis for all-cause readmission of HFrEF patients in relation to plasma catestatin concentration.

**Figure 3:**
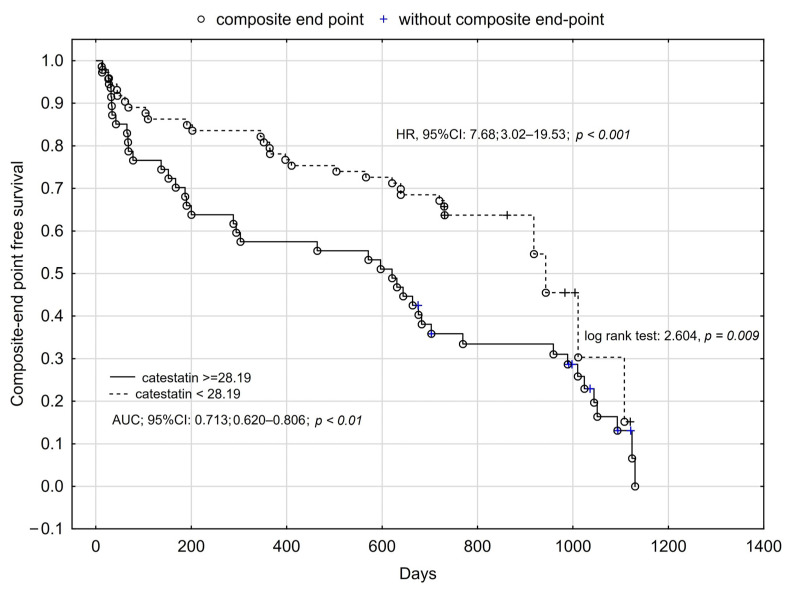
Kaplan–Meier analysis for composite endpoint of HFrEF patients in relation to plasma catestatin concentration.

Using cut-offs values obtained for catestatin ROC analysis for prediction of all-cause mortality, readmission, and composite endpoint occurrence, we performed Kaplan–Meier analysis, which confirmed the influence of catestatin on long-term prognosis among HFrEF patients. We found that higher plasma catestatin concentration increased the risk of all-cause death by more than five-times (Figure 1, Figure 2 and Figure 3).

**Figure 4:**
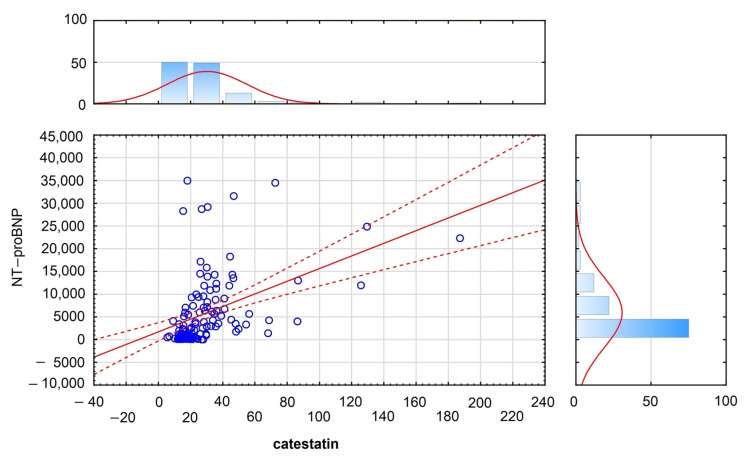
Correlation of catestatin and NT-proBNP, R = 0.439, *p* < 0.001. Individual data points represent Box-Cox transformed values. Solid red line—regression line estimated from the sample population. Red dashed lines—confidence curves = 0.95.

Using the Cox regression proportional hazard method, we tried to determine independent risk factors for measured outcomes among HFrEF patients. We achieved statistically significant regression models; however, none of the biomarkers included in the model reached statistical significance (Table 5, Table 6 and Table 7, Figure 4).

Annotation: X: catestatin; *n* = 120, mean = 29.813250; standard deviation = 24.573521; max = 187.230000; min = 5.520000; Y: NT-BNP; *n* = 120; mean = 5873.05000; standard deviation = 7789.3915; max = 35,000.00000; min = 24.000000; NT-BNP= 1724.8 + 139.14* catestatin; correlation: R = 0.43896; confidence interval = 0.95.

## 4. Discussion

Although all our patients were carefully qualified as having a reduced left ventricular ejection fraction, the presence of patients in various clinical conditions presenting both stable and exacerbated HFrEF courses reduced the homogeneity of the study group. Another limitation was their single ethnicity and the performance of the study in one research center, which reduced the representativeness of the tested marker for the world population. It should also be noted that the methodology used did not allow determining whether plasma CST concentration provided additional prognostic information compared to natriuretic peptides (NP), or whether adding CST to other known prognostic factors improved the prognostic model. However, the concentration of CST increased significantly with the NYHA class (R = 0.58; *p* < 0.001) and correlated significantly with the currently used markers of heart failure, including TNT (R = 0.22; *p* = 0.016) and NT-proBNP (R = 0.44; *p* < 0.001), but did not show a significant correlation with BMI (R = −0.0004; *p* = 0.997). According to information published in the 2021 ESC Guidelines for the diagnosis and treatment of acute and chronic heart failure [33,34], NP levels may be disproportionately low in obese individuals. Moreover, the cited guidelines emphasize that there are many causes of elevated NP levels, both cardiovascular and non-cardiovascular, that may reduce their diagnostic accuracy. Some examples of these are ischemic stroke, renal failure, cirrhosis, COPD, and anemia. Due to the differences in the correlation with BMI between CST and pro-BNP, the potential prognostic advantage of CST over NP should be the subject of future analyses in the obese population with the above diseases. CST is also not a specific marker for HFrEF, and other clinical situations with increased sympathetic activation in particular critical conditions including sepsis [18] may alter the prognostic value in the context of HF. Despite these limitations, our results indicate that elevated CST levels were associated with a worse prognosis among patients with HfrEF in the long-term follow-up. CST allows a statistically significant regression model, and the clinical usefulness of plasma CST concentration as a prognostic factor in HfrEF patients was not worse than NT-proBNP. Although CST did not reach statistical significance with regard to rehospitalizations, we found that a higher plasma CST concentration increased the risk of all-cause death by more than five times. The obtained data allowed identifying stable patients with the worst prognosis. Patients with high CST levels may benefit from systematic follow-up and may be candidates for more aggressive treatment. Changes in CST concentrations are the result of a number of related pathomechanisms involved in HF, such as oxidative stress, chronic inflammation, excessive activation of the immune system and sympathetic nervous system (SNS), and their consequences. Issues related to the pleiotropic effect of CST demonstrate that after the action of the factor leading to the change in the force of myocardial contraction, there must be a number of compensatory mechanisms that are interconnected and interdependent, and the circulatory system decompensates when a specific critical point is reached. CST is therefore part of a complex neurohumoral feedback system and is secreted as a counterregulatory peptide that attenuates excess catecholamines and SNS activity. Higher levels of this peptide in plasma may indirectly reflect increased neurohumoral load and dysfunctional baroreceptor control.

Since the incorporation of natriuretic peptide assessment into routine clinical practice, many reliable markers of heart failure have been tested with varying results. Considering the high residual risk, reflecting the multifaceted pathophysiology of heart failure, even with their significant clinical role, natriuretic peptides seem to be insufficient. Thus, a multi-marker approach may help to refine therapeutic strategies and allow treatment to be tailored to the individual clinical and biochemical profile of each patient with HF.

The analysis of the collected data suggests that higher plasma CST values in patients with HFrEF were associated with a worse prognosis in the long-term follow-up. Although the risks for CE, all-cause mortality, and all-cause rehospitalization depend on a constellation of various factors, CST provides a statistically significant regression model, which is why we consider CST to be a useful marker that is competitive with currently used markers of heart failure.

Our 2020 publication examining the CST level in the population of Polish patients with HFrEF showed that the difference in plasma CST concentration between healthy individuals and properly treated patients with stable CHF may be minimal or nonexistent [5].

Since CST reflects SNS mobilizations, in accordance with our assumptions, adding patients with an exacerbated clinical course of HFrEF and de novo HfrEF to the study group resulted in an increase in the CST level in correlation with the severity of NH symptoms according to the NYHA classification and a decrease in the LVEF value. This is because, like NT-proBNP, CST turns out to be a marker of hemodynamic instability. Available literature data on the usefulness of this marker during follow-up and the effect of pharmacotherapy on catestatin concentrations are minimal. A review of the current literature reveals four clinical studies examining plasma catestatin levels [5,14,35,36] and one examining serum [37] catestatin levels in various stages of HF.

Despite the use of a smaller and less homogeneous study group in terms of LVEF, conclusions similar to our findings were reached by Borovac et al. [37] in a non-randomized cross-sectional clinical trial conducted from January 2018 to February 2019. The study included 95 individuals with signs and symptoms of NYHA class II–IV HF. All patients were in a clinical exacerbation described as acute decompensated heart failure (ADHF), most with HFrEF (*n* = 39, 43.4%) and others with HFpEF (heart failure with preserved ejection fraction) (*n* = 31, 34.4%) or HFmrEF (heart failure with mid-range ejection fraction) (*n* = 20 22.2%). Contrary to other studies, Borovac et al. determined serum CST levels. In their multivariable linear regression analysis, CST independently correlated with NYHA class (β = 0.491, *p* < 0.001), waist-to-hip ratio (WHR) (β = −0.237, *p* = 0.026), HbA1c (β = −0.235, *p* = 0.027), LDL (β = −0.231, *p* = 0.029), non-HDL cholesterol (β = −0.237, *p* = 0.026), hs-cTnI (β = −0.221, *p* = 0.030), heart rate on admission, and resting heart rate (β = −0.201, *p* = 0.036 and β = −0.242, *p* = 0.030) and was associated with most echocardiographic parameters, but did not differ between patients with reduced, mild, or preserved LVEF (7.74 ± 5.64 vs. 5, 75 ± 4.19 vs. 5.35 ± 2.77 ng/mL, *p* = 0.143, respectively). ROC analysis showed that the plasma level of CST was comparable in terms of diagnostic efficacy and provided significantly higher AUC values in patients who died than in patients who survived compared to traditional biomarkers such as NT-proBNP and hs-cTnI [37]. Due to the compensatory effect of CST on adverse neurohumoral activation, which is especially pronounced in ischemic diseases, the researchers obtained significantly higher CST concentrations in patients with ADHF associated with myocardial infarction (MI) compared to patients without MI. Although the CST levels observed by Borovac et al. were not significantly different between the three LVEF phenotypes, the investigators noted a clear trend towards higher CST levels in HFrEF patients, although statistical significance was not reached, most likely due to limited sample size. In addition, circulating CST levels were highest in the NYHA IV subgroup, followed by NYHA III, and lowest in the NYHA II subgroup. This study is in line with our results. Other researchers (Liu et al.) [14] who included patients of all NYHA classes in their studies obtained similar concentrations in class I and II and significantly higher concentrations in class III and IV.

Only one of the available studies (Zhu et al.) [35], despite collecting the largest study group (*n* = 300), obtained a surprising (because inconsistent with other studies) inverse relationship between CST and clinical advancement of HF. The authors assessed CST levels in different phases of HF and the diagnostic utility of CST as a potential biomarker for the detection of asymptomatic HF in stage B according to the American Heart Association (AHA). In the group of 300 patients (stage B: *n* = 76, age 68.58 ± 8.63, LVEF 54.95 ± 9.82%), it was shown that the concentration of CST decreased from stage A, through B, to C. The cut-off point for the CST stage B HF detection value was 19.73 ng/mL, with a sensitivity of 90% (higher than BNP in this study) and a specificity of 50.9%. Contrary to our results, Zhu et al. found no correlation between CST and BNP concentration (R = 0.107, *p* = 0.150 versus R = 0.61, *p* < 0.001). According to the authors, asymptomatic patients with stage B HF will benefit most from regular follow-up and therapeutic intervention; as observed in this group, a decrease in catestatin levels may precede full-blown HF.

Comparing the group from our study with Zhu et al.’s stage C (the most appropriate comparison in terms of the occurrence of symptoms and the largest representation in the study group), significant differences should be emphasized regarding the optimal pharmacological treatment (beta-blocker 90.83% vs. 86.2%, ACEi or sartan 81.6% vs. 72.4%, spironolactone or eplerenone 77.5% vs. no data). After increasing the size of the study group, which in the current study also includes patients with exacerbation of HF and with de novo HF, we obtained divergent conclusions to Zhu et al. and a positive correlation of CST with clinical severity of HF.

Although our study did not assess the effect of treatment on HF marker levels, interesting observations come from Liu et al. [14]. In their research, plasma CST concentrations did not decrease significantly despite treatment and alleviation of HF symptoms, while plasma BNP concentrations decreased significantly. This finding may suggest retention of residual catecholaminergic activity, which is not reflected in the reduction of circulating natriuretic peptides. Optimal pharmacological treatment should include further suppression of the sympathetic component until normalization of CST levels. The difference in concentration between markers may also result from distinct mechanisms underlying the production, distribution and excretion of both markers. Simultaneous measurement of different markers may provide broader insight into different aspects of HFrEF pathophysiology and increase the prognostic value of the tests performed, which should be the basis of modern risk stratification.

Even if our analysis of patients with an exacerbation included clinically stable individuals, which reduced the homogeneity of the study group, the results obtained allowed us to isolate stable patients with the worst prognosis, which allowed for the early application of more aggressive treatment methods.

Future studies should focus on measuring CST among similar cohorts of HF patients, taking into account their clinical parameters, variability of CgA metabolism, medical history and pharmacotherapy (including new-generation drugs such as levosimendan [38] and proprotein convertase subtilisin/kexin 9 inhibitors [39]). Such actions will make it possible to estimate the range of norms of the persistent sympathetic component in patients with HF, assess the effectiveness of treatment and direct further pharmacotherapy to the residual neurohormonal disease.

The limitations of the present study were the relatively small study population and the lack of assessment of possible changes in chromogranin A proteolysis disorders in the study group.

## 5. Conclusions

The prognostic model for the study endpoints—unplanned hospitalization, all-cause death, and the composite endpoint—depends on a constellation of different factors. Higher plasma catestatin concentration was associated with worse prognosis in HFrEF patients during long-term follow-up and increased the risk of all-cause death by more than five times. Plasma CST concentration increased significantly with NYHA class (R = 0.58; *p* < 0.001) and correlated significantly with currently used HF markers, including TNT (R = 0.22; *p* = 0.016) and NT-proBNP (R = 0.44; *p* < 0.001). Patients with high CST levels may benefit from systematic follow-up and may be candidates for more aggressive treatment. Due to the differences in the correlation with BMI between CST and NT-proBNP and the disproportionately low levels of natriuretic peptides in the obese population, future studies should evaluate the possible prognostic advantage of CST in overweight cardiac patients.

## Figures and Tables

**Table 1:** Comparison of baseline parameters studied in patients who died or survived during long-term follow-up.

Parameter (Unit)	Death (*n* = 44)	Survived (*n* = 76)	*p*
Male sex	34 (77.27)	59 (77.63)	0.964
Age (years)	69.91± 13.74	55.53± 12.80	<0.001
NYHA (II. III. IV)	2 (4.55) 17 (38.64) 25 (56.82)	37 (48.68) 26 (34.21) 13 (17.11)	<0.001
Readmission (%)	27 (61.36)	26 (34.21)	0.004
TAPSE (mm)	16.89 ± 3.90	19.64 ± 4.80	<0.001
EF (%)	24.82 ± 8.63	27.88 ± 8.28	0.057
Etiology (DCM/ICM)	20 (45.45) 24 (54.55)	50 (65.79) 26 (34.21)	0.030
BMI (kg/m^2^)	28.39 ± 5.93	29.87 ± 5.99	0.194
DM	19 (43.18)	30 (39.47)	0.693
HT	26 (59.09)	37 (48.68)	0.275
AF	27 (61.36)	32 (42.11)	0.390
ICD/CRT-d	26 (58.09)	62 (61.58)	0.005
NT-proBNP (pg/mL)	9620.80 ± 8883.64	3703.30 ± 6165.45	<0.001
Catestatin (ng/mL)	38.94 ± 33.30	24.53 ± 15.66	0.002
TNT (μg/L)	0.06 ± 0.07	0.03 ± 0.05	0.003
Creatinine (mg/dL)	1.40 ± 0.46	1.09 ± 0.32	<0.001
hsCRP (mg/L)	16.91 ± 27.43	11.55 ± 30.01	0.333
HB (g/dL)	12.45 ± 2.40	14.07 ± 1.66	<0.001
HCT (%)	37.94 ± 7.18	41.63 ± 4.26	<0.001
PLT (1000/mm^3^)	182.39 ± 72.27	211.20 ± 68.02	0.031
RDW (%)	16.65 ± 2.86	14.38 ± 1.87	<0.001
WBC (1000/mm^3^)	8.71 ± 2.91	7.74 ± 2.25	0.043
NEUT (1000/mm^3^)	7.21 ± 5.22	4.84 ± 1.81	<0.001

TAPSE: tricuspid annular plane systolic excursion; EF: ejection fraction; DCM/ICM: dilated cardiomyopathy/ischemic cardiomyopathy; BMI: body mass index; DM: diabetes mellitus; HT: hypertension, AF: atrial fibrillation; ICD/CRT-d: implantable cardioverter-defibrillator/cardiac resynchronization therapy with defibrillator; NT-proBNP: N-terminal fragment of B-type natriuretic propeptide; TNT: troponin t; hsCRP: high-sensitivity CRP; HB: hemoglobin; HCT: hematocrit; PLT: platelets; RDW: red cell distribution width; WBC: white blood cells; NEUT: neutrophils.

**Table 2:** Differences regarding received pharmacotherapy among patients who died or survived throughout long-term follow-up.

Parameter (Unit)	Death (*n* = 44)	Survived (*n* = 76)	*p*
ACEI	22 (50.00)	51 (67.11)	0.065
ARB	8 (18.18)	17 (22.37)	0.590
ASA	5 (11.36)	28 (36.84)	0.002
BB	39 (88.64)	70 (92.11)	0.530
Digoxin	5 (11.36)	8 (10.53)	0.888
Statin	35 (79.55)	58 (76.32)	0.686
Ivabradine	4 (9.09)	7 (9.21)	0.983
VKA	9 (20.45)	19 (25.00)	0.574
Amiodarone	8 (18.18)	6 (7.89)	0.092
NOAC	23 (52.27)	16 (21.05)	<0.001
Spironolactone	14 (31.82)	31 (40.79)	0.332
Eplerenone	13 (29.55)	35 (46.07)	0.076
Furosemide	8 (18.18)	22 (28.95)	0.192
Torasemide	29 (65.91)	28 (36.84)	0.002
HCTZ	2 (4.55)	8 (10.53)	0.257

ACEI: angiotensin-converting-enzyme inhibitors; ARB: angiotensin II receptor blockers; ASA: acetylsalicylic acid; VKA: vitamin K antagonist; NOAC: non-vitamin K antagonist oral anticoagulants; HCTZ: hydrochlorothiazide.

**Table 3:** Parameters studied in HFrEF patients in relation to plasma catestatin concentration.

Parameter (Unit)	Catestatin ≥ 27.94 (*n* = 47)	Catestatin < 27.94 (*n* = 73)	*p*
Male sex	34 (72.34%)	59 (80.82)	0.342
Age (years)	69.71 ± 14.42	55.08 ± 11.94	<0.001
NYHA (II. III. IV)	1 (2.13%) 22 (46.81%) 24 (51.06%)	38 (52.05) 21 (28.77) 14 (19.18)	<0.001
Readmission (%)	26 (55.32%)	27 (36.99)	0.048
All-causes death	26 (55.32%)	16 (21.92)	<0.001
Composite endpoint	39 (82.98%)	31 (42.47)	<0.001
Survival time (days)	587.53 ± 371.82	710.37 ± 202.32	0.02
TAPSE (mm)	16.51 ± 3.57	20.04 ± 4.01	<0.001
EF (%)	27.10 ± 9.25	26.82 ± 8.10	0.860
Etiology (DCM/ICM)	23 (48.94%) 24 (51.10%)	47 (64.38) 26 (35.62)	0.057
BMI (kg/m^2^)	29.51 ± 5.36	29.22 ± 6.33	0.789
DM	24 (51.06%)	25 (34.25)	0.066
HT	30 (68.83%)	33 (45.21)	0.029
AF	28 (59.57%)	30 (41.10)	0.003
ICD/CRTD	20 (42.55%)	66 (90.41)	<0.001
NT-proBNP (pg/mL)	9423.00 ± 7950.45	3405.51 ± 6609.79	<0.001
TNT (μg/L)	0.07 ± 0.08	0.02 ± 0.03	<0.001
Creatine (mg/dL)	1.41 ± 0.46	1.06 ± 0.30	<0.001
Glucose (mg/dL)	141.29 ± 46.15	120.01 ± 41.00	<0.01
hsCRP (mg/L)	17.40 ± 26.13	10.82 ± 30.44	0.219
HB (g/dL)	12.31 ± 2.17	14.19 ± 1.75	<0.001
HCT (%)	37.45 ± 5.85	42.03 ± 5.03	<0.001
PLT (1000/mm^3^)	197.73 ± 77.99	203.59 ± 65.44	0.655
RDW (%)	16.23 ± 2.59	14.57 ± 2.24	<0.001
WBC (1000/mm^3^)	8.56 ± 2.51	7.78 ± 2.54	0.096
NEUT (%)	70.90 ± 10.93	62.47 ± 11.81	<0.001
NEUT (1000/mm^3^)	6.03 ± 2.53	5.45 ± 4.19	0.389
ACEI	19 (40.43%)	54 (73.97)	<0.001
ARB	10 (21.27%)	15 (20.55)	0.804
ASA	10 (21.27%)	23 (31.51)	0.179
BB	38 (80.85%)	71 (97.26)	<0.01
Digoxin	4 (8.51%)	9 (12.33)	0.469
Statin	35 (74.47%)	58 (79.54)	0.611
Ivabradine	3 (6.38%)	8 (10.96)	0.365
VKA	7 (14.89%)	21 (14.29)	0.063
NOAC	24 (51.06%)	15 (53.06)	<0.001
Amiodarone	8 (17.02%)	6 (8.22)	0.171
Spironolactone	18 (38.30%)	27 (36.99)	0.843
Eplerenone	6 (12.77%)	42 (57.53)	<0.001
Furosemide	10 (21.27%)	20 (27.40)	0.542
Torsemide	28 (59.57%)	29 (39.73)	0.034
HCTZ	1 (2.13%)	9 (12.33)	0.043

NYHA: New York Heart Association Functional Classification; TAPSE: tricuspid annular plane systolic excursion; EF: ejection fraction; DCM: dilated cardiomyopathy; ICM: ischemic cardiomyopathy; BMI: body mass index; DM: diabetes mellitus; HT: hypertension AF: atrial fibrillation; ICD/CRTD: implantable cardioverter-defibrillator cardiac resynchronization therapy with defibrillator; NT-proBNP: N-terminal fragment of prohormone B-type natriuretic propeptide; TNT: troponin t; hsCRP: high-sensitivity CRP; HB: hemoglobin; HCT: hematocrit; PLT: platelets; RDW: red cell distribution width; WBC: white blood cells; NEUT: neutrophils; ACEI: angiotensin-converting-enzyme inhibitors; ARB: angiotensin II receptor blockers; ASA: acetylsalicylic acid; BB: beta adrenergic receptor antagonists; VKA: vitamin K antagonist; NOAC: non-vitamin K antagonist oral anticoagulants; HCTZ: hydrochlorothiazide.

**Table 4:** Correlations between catestatin and selected parameters, *n* = 120.

Parameter (Unit)	Correlations between Catestatin and Selected Parameters	
	r	*p*
Age (years)	0.3711	<0.001
EF (%)	−0.0663	0.472
BMI (kg/m^2^)	−0.0004	0.997
TAPSE (mm)	−0.2793	0.002
NT-proBNP (pg/mL)	0.4390	<0.001
TNT (μg/L)	0.2195	0.016
Creatinine (mg/dL)	0.2937	0.001
hsCRP (mg/L)	0.1120	0.223
Glucose (mg/dL)	0.1970	0.031
HB (g/dL)	−0.2483	0.006

EF: ejection fraction; BMI: body mass index; TAPSE: tricuspid annular plane systolic excursion; NT-proBNP: N-terminal fragment of prohormone B-type natriuretic propeptide; TNT: troponin t; hsCRP: high-sensitivity CRP; HB: hemoglobin.

**Table 5:** Cox proportional hazard regression model for death prediction, chi^2^ = 48.69; *p* < 0.001.

Parameter (Unit)	HR	*p*
NT-proBNP (pg/mL)	1.00; 1.00–1.00	0.437
Catestatin (ng/mL)	1.004; 0.994–1.014	0.414
Creatinine (mg/dL)	1.791; 0.942–3.406	0.076
Age (years)	1.054; 1.025–1.085	<0.001
BMI (kg/m^2^)	0.924; 0.864–0.988	0.021
LVEF (%)	0.950; 0.912–0.990	0.014

NT-proBNP: N-terminal fragment of prohormone B-type natriuretic propeptide; BMI: body mass index; LVEF: left ventricle ejection fraction.

**Table 6:** Cox proportional hazard regression model for prediction of all-cause readmission, chi^2^ = 26.51; *p* < 0.001.

Parameter (Unit)	HR. 95%CI	*p*
NT-proBNP (pg/mL)	1.00; 1.00–1.00	0.047
Catestatin (ng/mL)	1.003; 0.99–1.02	0.664
Creatinine (mg/dL)	1.58; 0.85–2.94	0.153
Age (years)	1.03; 1.01–1.05	0.018
BMI (kg/m^2^)	0.97; 0.92–1.03	0.315
LVEF (%)	0.96; 0.93–0.99	0.047

NT-proBNP: N-terminal fragment of prohormone B-type natriuretic propeptide; BMI: body mass index; LVEF: left ventricle ejection fraction.

**Table 7:** Cox proportional hazard regression model for prediction of composite endpoint, chi^2^ = 48.29; *p* < 0.001.

Parameter (Unit)	HR. 95%CI	*p*
NT-proBNP (pg/mL)	1.00; 0.99–1.00	0.151
Catestatin (ng/mL)	1.01; 0.99–1.01	0.316
Creatinine (mg/dL)	1.48; 0.85–2.56	0.163
Age (years)	1.04; 1.02–1.06	<0.001
BMI (kg/m^2^)	0.95; 0.91–1.00	0.063
LVEF (%)	0.95; 0.92–0.98	0.004

NT-proBNP: N-terminal fragment of prohormone B-type natriuretic propeptide; BMI: body mass index; LVEF: left ventricle ejection fraction.

## Data Availability

All data used to support the finding of this study are available from the corresponding author upon request.
